# Computational Framework for Structuring and Analyzing Clinical Trial Criteria for AI-Guided Fine-grained Matching

**DOI:** 10.1007/s10916-025-02303-y

**Published:** 2025-11-22

**Authors:** Daniel R. S. Habib, Ishan Mahajan, Betina Evancha, Christine Micheel, Daniel Fabbri

**Affiliations:** 1 Vanderbilt University School of Medicine, Nashville, TN USA; 2 Vanderbilt University, Nashville, TN USA; 3 Vanderbilt-Ingram Cancer Center, Nashville, TN USA; 4 Department of Biomedical Informatics, Vanderbilt University Medical Center, 1211 Medical Center Dr, Nashville, TN USA; 5 Vanderbilt University School of Engineering, Nashville, TN USA

**Keywords:** Artificial intelligence, Eligibility determination, Natural language processing, Medical informatics, Decision support systems, Clinical

## Abstract

While artificial intelligence (AI) has demonstrated potential in automating clinical trial matching, most existing solutions rely on high-level structured data or oversimplified criteria. This study introduces a framework to structure and analyze eligibility criteria across three real-world trial protocols, aiming to inform more granular AI-driven trial matching strategies. Trial criteria from three protocols were decomposed into individual variables and evaluated based on data type, scope, and dependency. Complexity was assessed using a novel formula incorporating the number of independent and dependent variables, alongside the Flesch-Kincaid reading grade level. Quantitative analysis explored variation across trials. Protocols contained between 22–160 eligibility variables, with 4–22% showing interdependence. Reading grade levels ranged from sixth grade to first-year college. Complexity scores varied significantly, with some trials exhibiting particularly high cognitive and logical burdens. Recursive and hierarchical structures were prevalent in high-complexity protocols. This study reveals the substantial variability and structural complexity of clinical trial criteria, highlighting challenges for AI matching systems. A standardized approach to measuring trial complexity can enhance algorithm transparency, scalability, and interpretability. These findings underscore the need for structured, computable frameworks to improve equity and efficiency in clinical trial recruitment.

**Supplementary Information:**

The online version contains supplementary material available at 10.1007/s10916-025-02303-y.

## Introduction

Clinical trials are necessary for medical innovation, but recruitment remains a substantial obstacle [1, 2]. In 2020, 41% of Americans reported not knowing anything about clinical trials, and only 5% of eligible adult cancer patients participated in trials [3, 4]. Four of the primary enrollment barriers include healthcare professional time constraints, limited awareness of available trials, strict eligibility criteria, and complex clinical design [5]. Additional barriers, such as logistical challenges and historical mistrust, further impede participation, especially among underrepresented populations [6, 7]. These challenges are particularly prominent in rare disease research, where eligible patient populations are inherently limited and geographically dispersed [8]. Low enrollment is the main reason for randomized controlled trials (RCTs) stopping early [9], which compromises statistical power, yields inconclusive results, and wastes resources [10]. Safely lowering trial participation barriers would improve the accuracy and speed of data collection on new treatments, facilitating drug safety and timely release to the public [11]. With drug development costs exceeding $2.6 billion apiece due in part to inefficiencies in the clinical trial process [12], innovative approaches are needed to streamline matching while addressing accessibility, transparency, and inclusivity.

**Fig. 1:**
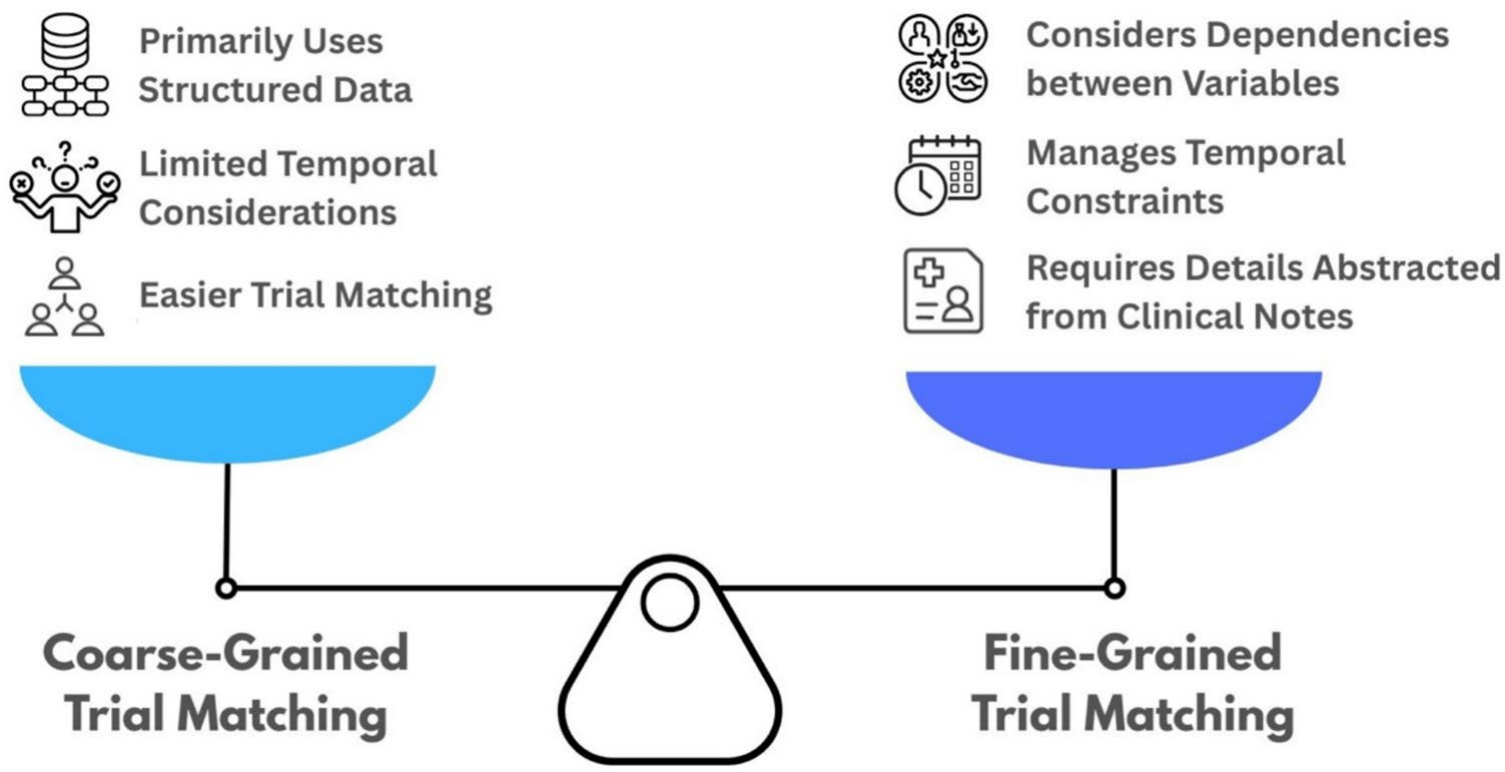
Characteristics of coarse-grained and fine-grained clinical trial matching

Artificial intelligence (AI) has garnered significant attention in clinical trial matching. Coarse-grained matching, which uses higher-level structured criteria (e.g., International Classification of Diseases [ICD] codes), enables subgroup analysis but is insufficient for trials with more detailed or unstructured eligibility requirements [13]. For example, TrialGPT matched patients to potential trials with 87% accuracy, decreasing screening time by 43%, but relied on high-level criteria [14]. Oncology trials increasingly rely on intricate biomarker-driven eligibility, demanding advanced frameworks that integrate molecular, clinical, and demographic parameters [15]. Fine-grained matching requires extracting and aggregating multiple data points, often from unstructured notes, and applying logical or temporal relationships (Fig. 1). Moreover, the criteria include logical and/or temporal functions across multiple extracted data points to determine eligibility. These fine-grained criteria require multi-stage analyses across many clinical notes of different formats and hundreds of variables to be extracted per trial to include/exclude more specific groups of patients, allowing for more detailed hypothesis testing. For instance, confirming that “at least 12 months have elapsed between the last curative treatment and disease recurrence” requires extracting and reasoning over several timestamps and events. Recent work shows zero-shot large-language models (LLMs) can reduce time and cost for patient matching while maintaining high accuracy [16].

Although coarse-grained approaches may assist initial recruitment, many trials benefit from fine-grained automated prescreening. For example, tumor next-generation sequencing (NGS) reports may inform trial recommendations, but prescreening is required to verify eligibility; manual prescreening after biomarker-driven recommendations reduced physician burden and false positives [17]. AI-assisted screening tools like Retrieval-Augmented Generation (RAG)-Enabled Clinical Trial Infrastructure for Inclusion Exclusion Review (RECTIFIER) not only reduce screening time but also improve enrollment rates, as shown in heart failure clinical trials [18]. However, there remains no framework for creating and assessing the complexity of fine-grained clinical trial matching. This paper presents a computational framework for structuring and analyzing clinical trial criteria to enable AI-guided fine-grained matching. We define units of computation, introduce a complexity scoring system, and present real-world use cases across three clinical trials to demonstrate the practical application of our approach.

## Methods

Three real clinical trial protocols were collected from a large academic hospital. The three analyzed trial protocols represented distinct therapeutic domains: oncology (DTBRE23078, Phase 3 RCT), precision medicine (MATCH, Phase 2 basket trial), and observational cardiology (INSIGHT). Each protocol’s inclusion and exclusion criteria were extracted directly from the official protocol documents rather than operational checklists, ensuring that the structured decomposition reflected the authoritative source text used in trial design.

**Fig. 2:**
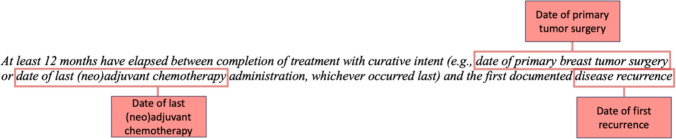
Example of manually translating unstructured clinical trial criterion free text into independent variables

Protocols were mapped to variables and associated data elements for extraction to determine eligibility. From the example above, variables include date of surgery, date of last (neo)adjuvant chemotherapy, and date of recurrence. An independent variable is defined as a discrete unit of data extracted from clinical text based on provided logical instructions. Computationally, a variable operates as a RAG on top of clinical notes with instructions. Figure 2 shows an example of the independent variables defined to capture the data points needed to calculate time between definitive treatment and recurrence. The discrete unit of data here represents the timestamps of events.

Many independent variables can be collated to capture more complex trial criteria. A dependent variable takes as input one or more independent or dependent variables, and using logical instructions, extracts a discrete unit of data. Unlike an independent variable, dependent variables do not read directly from clinical text, only taking results from variables as inputs. The recursive definition of dependent variables, where they can be used as input to other dependent variables, allows for the representation of complex clinical trial criteria through a hierarchy.

Variables are further categorized by data type and scope (Table 1). Data types include standard programming types such as integer, float, boolean, timestamp, or text. Indeterminate was defined as not falling into a standard data type (e.g., eligibility based on clinician intuition). The scope captures the granularity by which data should be aggregated across notes. In the simplest case, data are not aggregated and have a scope as many-values-per-note. Additionally, data can be aggregated within a note (one-value-per-note) or aggregated across a patient (one-value-per-patient). For example, a variable that captures if a patient ever has cancer would have a scope of one-value-per-patient, while a variable that captures each medication administration would have a scope of many-values-per-note. A range of aggregation strategies can be considered for one-value-per-patient including choosing the most frequent value, the first value, the last value, the earliest date of occurrence, the latest date of occurrence, and others.

**Table 1:** Explanations of variable attributes: data type, scope, and dependency

Variable Attributes	Explanation
*Data Type*	Standard data formats such as integer, float, boolean, timestamp, or text. "Indeterminate" for non-standard cases (e.g., eligibility based on clinical intuition)
*Scope*	Defines how data are aggregated across notes
Many Per Note	Variable appears multiple times within a single clinical note (e.g., medication administrations)
One Per Note	Variable is aggregated to a single value within a single clinical note (e.g., highest recorded temperature in a daily progress note)
One Per Patient	Variable is aggregated across multiple notes for a patient (e.g., whether a patient has ever had cancer)
*Dependency*	Defines whether a variable is independent or dependent
Independent	Extracted directly from clinical text using logical instructions (e.g., date of primary tumor surgery)
Dependent	Computed based on independent or other dependent variables using logical operations, without direct text extraction (e.g., time between definitive treatment and first cancer recurrence)

Using this design, clinical trial eligibility is defined as the boolean output of a dependent variable, where the dependent variable may have multiple independent and dependent variable inputs. In practice, the clinical trial eligibility is determined by collating the output from multiple variables, where trial criteria would ultimately be represented by dependent variables. In the case of the three analyzed clinical trials, hundreds of variables are defined to determine eligibility. Domain experts, including clinical research coordinators and investigators, were consulted during the decomposition process to validate variable mappings and ensure clinical relevance. Examples of these clinical trial mappings are provided in the results section.

**Fig. 3:**
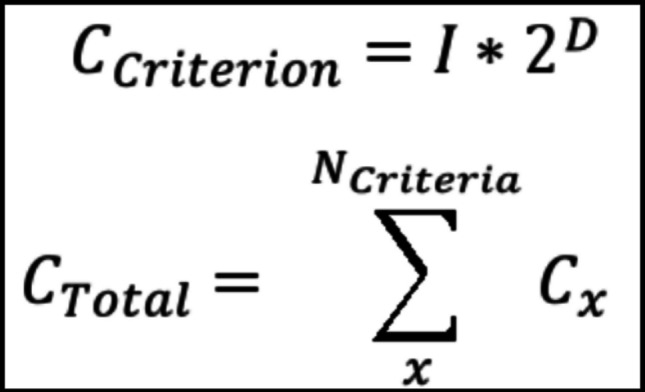
Formulas for criterion and total complexity scoring. C: Complexity; I: Number of unique independent variables; D: Number of dependent variables

To measure clinical trial complexity, two metrics were used. Flesch-Kincaid reading grade level considers the number of syllables, words, and sentences to approximate the U.S. grade level required to understand a piece of text (https://storytoolz.com/readability). Criterion complexity is defined as the number of unique independent variables times 2 to the power of the number of dependent variables (Fig. 3). The total trial complexity for a trial protocol equals the sum of criteria complexity scores for that trial.

While Flesch-Kincaid level estimates ease of reading for a human clinical trial screener, trial complexity quantifies the computational challenge of automatically screening patients. Independent variable retrieval involves finding data points while dependent variable retrieval requires combining variables and thus contributes a higher level of complexity. To understand how these two measures are related, Spearman’s rank correlation coefficients were calculated for complexity by word count and Flesch-Kincaid reading grade level.

## Results

**Fig. 4:**
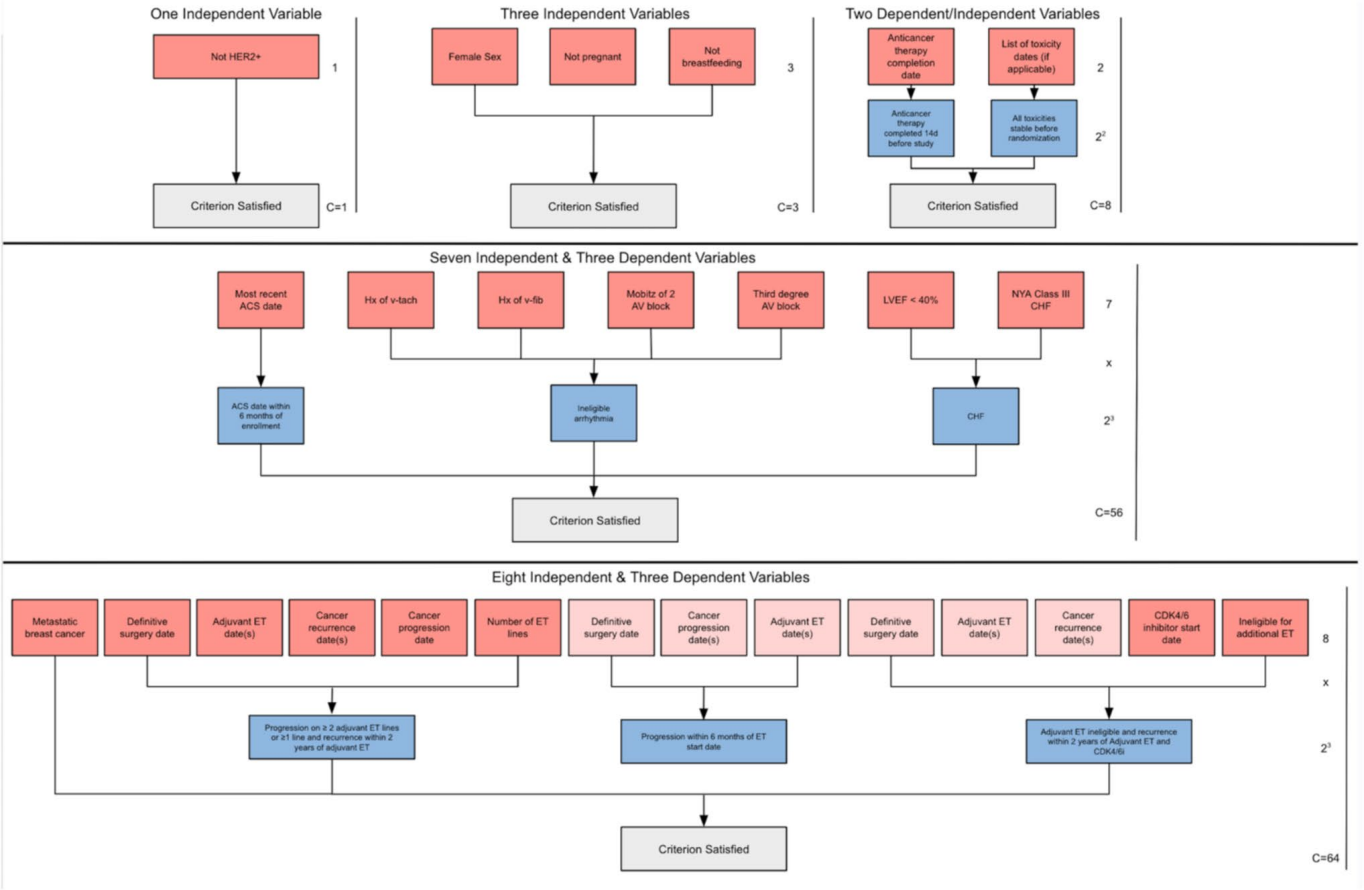
Top-down example hierarchies of independent (red) and dependent (blue) variables as well as complexity scores for five criteria from one clinical trial. ACS: acute coronary syndrome; CHF: congestive heart failure; Hx: history; V-tach: ventricular tachycardia; V-fib: ventricular fibrillation; AV: atrioventricular; LVEF: left ventricular ejection fraction; NYHA: New York Heart Association; ET: endocrine therapy; CDKi: cyclin-dependent kinase inhibitor

Three clinical trial protocols (A-C) contained 8 to 39 criteria (Table 2). Median reading grade levels for each criterion ranged from sixth grade to first year of college. Trial A exhibited a high word count and reading level. Trial B contained full sentences but with simpler language. Trial C contained predominantly bullet points with even simpler language. The three protocols contained 22 to 159 variables. Boolean was the predominant data type. The scope of most variables was one-per-patient (e.g., age). Dependent variables ranged from 4 to 22% of the total number of variables needed to define the trial criterion. Figure 4 illustrates examples of how independent and dependent variables were structured to represent clinical trial criteria, which are explained in more detail below.

**Table 2:** Sentence-level and variable-level characteristics for three clinical trials

	Trial A	Trial B	Trial C
Number of Criteria	39	14	8
Word Count			
Median (Interquartile Range)	27 (13–54)	16 (10–23)	9 (6–15)
Total	1605	203	82
Reading Level Per Criterion			
Median (Interquartile Range)	13.1 (9.1–15.7)	12.0 (10.4–14.4)	5.9 (4.5–9.2)
Highest	33.5	19.6	9.5
Total Number of Variables	160	26	22
Variable Data Type			
Integer	2	0	4
Float	2	0	0
Boolean	111	17	16
Text	14	0	0
Timestamp	24	3	2
Indeterminate	7	6	0
Variable Scope			
Many Per Note	4	3	0
One Per Note	3	8	0
One Per Patient	153	15	22
Variable Dependency			
Independent	131	25	19
Dependent	29	1	3
Complexity			
Median (Interquartile Range)	2.0 (1.0–8.0)	3.0 (2.0–4.0)	2.0 (1.0–2.5)
Total	496	31	30

The first example text is: Not previously HER2 + on pathology testing. The text contains one independent variable and no dependent variables. The complexity equals 1.

The second example text is: Does not have a positive serum pregnancy test and is not breastfeeding for patients who are assigned female at birth. The text contains three independent variables (each of which returns the most recent values) and no dependent variables. The complexity equals 3.

The third example text is: Patients must have completed any anticancer treatment greater than or equal to 14 days prior to randomization. Any toxicity experienced on prior treatment must have resolved or be considered clinically stable prior to randomization. The text contains two independent variables (each of which returns the most recent values) and two dependent variables. The complexity equals 2 (independent variables) times 2 to the power of 2 (dependent variables), which equals 8.

The fourth example text is: Patient must not have a history of significant cardiovascular disease, defined as: a) Myocardial infarction or unstable angina pectoris within 6 months of enrollment. b) History of serious ventricular arrhythmia (i.e., ventricular tachycardia or ventricular fibrillation), high-grade atrioventricular block, or other cardiac arrhythmias requiring antiarrhythmic medications (except for atrial fibrillation that is well controlled with antiarrhythmic medication); history of QT interval prolongation. c) New York Heart Association Class III or greater congestive heart failure or known left ventricular ejection fraction of < 40%*.* This criterion is composed of three sub-criteria. In total, there are seven independent variables (each of which returns the most recent value) and three dependent variables. The complexity equals 7 (independent variables) times 2 to the power of 3 (dependent variables), which equals 56.

The fifth example text is: Patients must have one of the following: a) Disease progression on greater than or equal to 2 or more previous lines of ET with or without a targeted therapy in the metastatic setting. Disease recurrence while on the first 24 months of starting adjuvant ET will be considered a line of therapy; these patients will only require 1 line of ET in the metastatic setting. b) Disease progression within 6 months of starting first-line ET with or without a CDK 4/6 inhibitor in the metastatic setting. c) Disease recurrence while on the first 24 months of starting adjuvant ET with CDK 4/6 inhibitor and if the patient is no longer a candidate for additional ET in the metastatic setting*.* This criterion is composed of three sub-criteria. In total, there are eight unique independent variables (four of which are used multiple times) and three dependent variables. Six independent variables return the most recent value while the other two return multiple dates as applicable. The complexity equals 8 (unique independent variables) times 2 to the power of 3 (dependent variables), which equals 64.

**Fig. 5:**
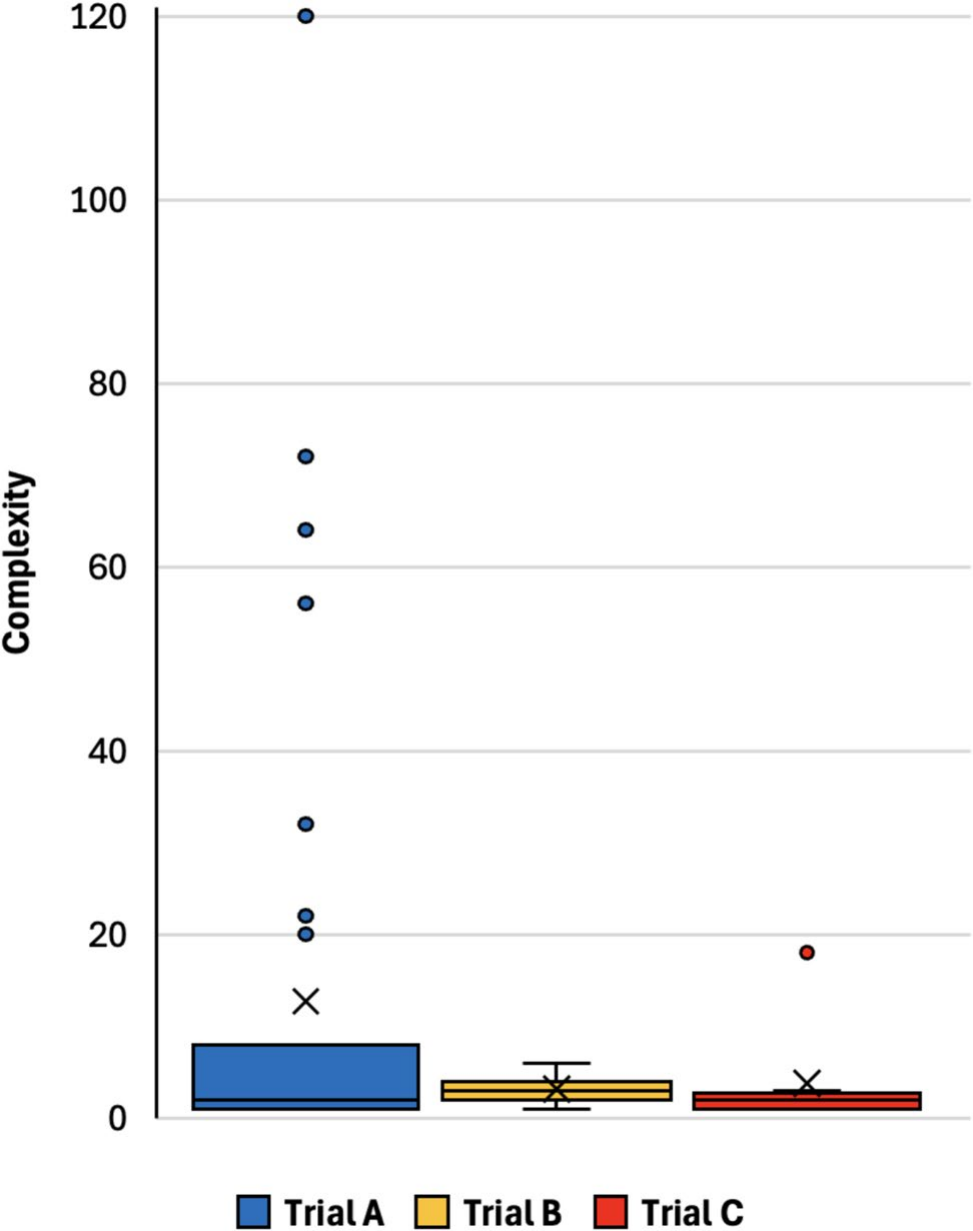
Box and whisker plots of complexity scores for each criterion in three clinical trials. Bottom and top of each box indicate lower and upper quartiles. The inner line indicates the median while “X” marks the average. Dots indicate outliers that are 3/2 times the upper or lower quartile while upper and lower whiskers indicate the highest and lowest values, excluding outliers

The median (IQR) criterion complexity of Trials A, B, and C across all criteria was 2.0 (1.0–8.0), 3.0 (2.0–4.0), and 2.0 (1.0–2.5), respectively. As Fig. 5 shows, a subset of criteria has extremely high complexity. The total complexity for Trials A-C was 496, 31, and 30, respectively. The association of complexity with word count and reading level varied by the way criteria were written for each of the trials. Complexity was strongly correlated with word count for Trials A and B but not C while complexity exhibited non-significant trends toward weak to moderate correlation with reading level for Trials A and B but not C (Table 3).

**Table 3:** Spearman’s rank correlation coefficients for complexity by word count and Flesch-Kincaid reading grade level

	Trial A	Trial B	Trial C
Word Count	rho = 0.76; *p* < 0.001	rho = 0.79; *p* = 0.007	rho = 0.08; *p* = 0.860
Flesch-Kincaid Reading Grade Level	rho = 0.28; *p* = 0.090	rho = 0.50; *p* = 0.143	rho = 0.37; *p* = 0.374

Spearman’s rho ranges from 1.0 (perfect positive correlation) to 0 (no correlation)

## Discussion

The three analyzed clinical trials demonstrated varying levels of complexity, with boolean data type and one-per-patient scope dominating. Independent and dependent variables interact in a multi-stage hierarchy to satisfy eligibility requirements. The proportion of dependent to independent variables differed substantially between trial protocols. Although complexity scores for most criteria were relatively low, several criteria for each trial exhibited substantially higher complexity due to a large number of independent variables or the presence of dependent variables. The median reading grade levels of trial criteria ranged from sixth grade to first-year college level.

The three clinical trials (A–C) analyzed included DTBRE23078, MATCH, and INSIGHT. For example, DTBRE23078 is a Phase 3 randomized-controlled trial investigating Sacituzumab Govitecan versus physician-selected treatment in HR +/HER2- advanced breast cancer [19]. The eligibility criteria documented internally in PDFs, like those sourced from OnCore, often differ significantly from publicly available summaries on ClinicalTrials.gov. Internal PDFs typically present more detailed and explicit instructions, variable definitions, and specific clinical conditions required for enrollment, reflecting comprehensive protocol specifics and often including confidential or proprietary trial elements not fully listed online. In contrast, ClinicalTrials.gov tends to provide higher-level criteria summaries, ultimately omitting necessary methodological guidance or nuanced eligibility conditions. This high level of abstraction in publicly available summaries underscores the need for careful cross-referencing to ensure completeness and accuracy when developing automated matching algorithms.

A critical question in AI-guided clinical trial matching is the extent to which human expertise versus AI automation defines the structure, hierarchy, and definitions of eligibility criteria [20]. Traditionally, humans review clinical trial protocols and manually establish the multi-level structure of inclusion and exclusion criteria. Inclusion criteria often follow conjunctive (AND) logic, while exclusion criteria are frequently structured with disjunctive (OR) logic. This distinction is important for computational parsing, as AI systems must handle these logical operators differently to preserve clinical validity. Failure to account for this difference may bias eligibility assessments. While manual review ensures clinical nuance, it is labor-intensive and prone to variability. AI can assist by automating the extraction and hierarchical organization of eligibility criteria from unstructured documents and systematically analyzing patient data against this structured framework. The ability to first define a structured representation of trial criteria is critical, as it allows AI to analyze eligibility more systematically, reducing inconsistencies and improving efficiency.

To enable problem identification and resolution while safeguarding data privacy and appropriate use, LLM deployment in trial matching necessitates transparency and standardization. The TRIPOD-LLM reporting guideline underscores the need for structured methodologies when LLMs are applied in clinical settings to ensure reliability, explainability, and compliance in patient recruitment [21]. Jain et al. [15] proposes a conceptual framework that integrates electronic health records (EHRs), real-time patient data, and AI-driven analytics to streamline prescreening and enhance patient engagement. However, there remains no framework for creating and assessing the complexity of fine-grained clinical trial matching.

Ultimately, the optimal approach to patient matching may lie in a hybrid model: AI rapidly processes unstructured trial data and identifies discrete elements, while human experts refine and validate these results. A key challenge lies in AI’s ability to not only extract relevant data elements but also interpret their hierarchical and recursive dependencies within trial criteria. Many eligibility rules involve multi-step logic, requiring AI to understand how different conditions interact over time. Although we did not directly test AI model performance, our complexity framework has implications for AI-guided trial matching. High complexity scores may correlate with increased runtime and error rates in automated systems. Prior work supports combining human and machine intelligence to handle this complexity [22, 23]. Future work should explore strategies to enhance AI’s ability to autonomously define and analyze these dependencies, reducing the need for manual rule-setting while maintaining clinical accuracy. Additionally, developing scoring systems to quantify the complexity of trial-matching tasks will be crucial. By addressing these challenges, AI can move beyond simple, coarse-grained matching and advance toward a more sophisticated, clinically meaningful approach to trial eligibility assessment.

This study has limitations. First, the analysis was limited to three trials from a single institution, which constrains generalizability. Second, manual decomposition introduces subjectivity, as variable definitions and dependencies may differ across reviewers. Third, readability formulas such as Flesch-Kincaid capture linguistic complexity but do not fully reflect the cognitive burden for clinicians. Additionally, the formula to calculate criterion complexity may require further validation and refining by incorporating more context and variable characteristics such as variable type. Finally, overly granular decomposition risks excluding eligible patients when EHR data are incomplete or inconsistently coded, which may disproportionately affect underrepresented populations. Balancing precision with inclusivity remains a critical challenge.

## Conclusion

This study discusses challenges of fine-grained clinical trial matching. However, before that is possible, trial criteria must first be broken down into structured hierarchies of discrete elements of patient data. First, the paper defines the problem of clinical trial matching with fine-grained criteria, highlighting the recursive dependencies and multi-stage logic. Second, the computation units of clinical trial matching were defined using variables and dependent variables. Third, a novel yet simple complexity scoring system was introduced to quantify the computational burden of trial matching. Finally, these methods were applied to real-world use cases, analyzing three clinical trials of varying complexity to showcase trial criteria design and complexity. Human mapping of clinical trial variables informs future work transitioning from manually screening patients using complex protocol documents to automatically creating variable hierarchies using AI-guided chart abstraction and performing fine-grained matching.

## Supplementary Information

Below is the link to the electronic supplementary material.

Supplementary file1 (PDF 2.55 MB)

## Data Availability

Data is provided as part of the supplementary information files.
