# Supplementary material for: Computational Framework for Structuring and Analyzing Clinical Trial Criteria for AI-Guided Fine-grained Matching
Source: J Med Syst. 2025 Nov 22;49(1):168. doi: 10.1007/s10916-025-02303-y (PMC12640310; doi:10.1007/s10916-025-02303-y)
Supplement: Supplementary file 1 — Supplementary file1 (PDF 2.55 MB) [file 10916_2025_2303_MOESM1_ESM.pdf]

# STUDY SUBJECT ELIGIBILITY CHECKLIST

| | |
|---|---|
| **VICC Number and Study Title** | URO 20124 SPLASH: **S**tudy Evaluating Metastatic Castrate Resistant **P**rostate Cancer Treatment Using $^{177}$**L**u PNT2002 PSMA Therapy **A**fter **S**econd line **H**ormonal Treatment |
| **Protocol Version & Date** | Protocol Version 4.0 2022NOV23 |
| **Subject Initials** (First, Middle, Last) | |
| **Subject Medical Record Number** | |
| **Subject Date of Birth** | |

Patients must meet all of the following inclusion and exclusion criteria to be eligible for participation in this study. Refer to protocol to verify baseline test.

## Inclusion Criteria

(**Reminder:** All answers must be answered Yes or N/A for the subject to be eligible.)

| Yes | No | N/A | Inclusion Criteria | Criteria Answer | Date Criteria Completed |
|---|---|---|---|---|---|
| | | | 1. Male aged 18 years or older. | | |
| | | | 2. Histological, pathological, and/or cytological confirmation of adenocarcinoma of the prostate. | | |
| | | | 3. Ineligible or averse to chemotherapeutic treatment options. | | |
| | | | 4. Patients must have progressive mCRPC at the time of consent based on at least 1 of the following criteria:<br><br>a. Serum/plasma PSA progression defined as increase in PSA greater than 25% and >2 ng/mL above nadir, confirmed by progression at 2 time points at least 3 weeks apart.<br><br>b. Soft tissue progression defined as an increase ≥20% in the sum of the diameter (SOD) (short axis | | |

| | | | | | |
|---|---|---|---|---|---|
| | | | for nodal lesions and long axis for non-nodal lesions) of all target lesions based on the smallest SOD since treatment started or the appearance of one or a new lesion.<br><br>c. Progression of bone disease defined as the appearance of two or more new lesions by bone scan | | |
| | | | 5. Progression on previous treatment with one ARAT (abiraterone or enzalutamide or darolutamide or apalutamide) in either the CSPC or CRPC setting. | | |
| | | | 6. PSMA PET scan (i.e., $^{68}$Ga PSMA-11 or $^{18}$F-DCFPyL) positive as determined by the sponsor's central reader. | | |
| | | | 7. Castrate circulating testosterone levels (<1.7 nmol/L or <50 ng/dL). | | |
| | | | 8. Adequate organ function, independent of transfusion:<br><br>a. Bone marrow reserve:<br>i. White blood cell (WBC) count ≥ 2.5 × $10^9$/L OR absolute neutrophil count (ANC) ≥ 1.5 × $10^9$/L.<br><br>ii. Platelets ≥ 100 × $10^9$/L.<br><br>iii. Hemoglobin ≥ 8 g/dL.<br><br>b. Liver function:<br>i. Total bilirubin ≤ 1.5 × institutional upper limit of normal (ULN). For patients with known Gilbert's syndrome, ≤ 3 × ULN is permitted.<br><br>ii. ALT and AST ≤ 3.0 × ULN.<br><br>c. Renal function:<br>i. Serum/plasma creatinine ≤ 1.5 × ULN or creatinine clearance ≥ 50 mL/min based on Cockcroft-Gault formula (for patients in France, serum/plasma creatinine ≤ 1.5 × ULN or CrCl ≥ 60 mL/min based on | | |

| | | | | | |
|---|---|---|---|---|---|
| | | | Cockcroft-Gault formula).<br><br>d. Albumin ≥30 g/L. | | |
| | | | 9. Human immunodeficiency virus-infected patients who are healthy and have a low risk of acquired immunodeficiency syndrome-related outcomes are included in this trial. | | |
| | | | 10. For patients who have partners who are pregnant or of childbearing potential: a condom is required along with a highly effective contraceptive method during the study and for 6 months after last study drug administration. Such methods deemed highly effective include a) combined (estrogen and progestogen containing) hormonal contraception associated with inhibition of ovulation, b) progestogen-only hormonal contraception associated with inhibition of ovulation, c) intrauterine device (IUD), d) intrauterine hormone-releasing system (IUS), e) bilateral tubal occlusion, f) vasectomy, g) true sexual abstinence: when this is in line with the preferred and usual lifestyle of the subject [periodic abstinence (e.g., calendar, ovulation, symptothermal, post-ovulation methods), declaration of abstinence for the duration of exposure to IMP, and withdrawal are not acceptable methods of abstinence]. | | |
| | | | 11. Willing to initiate ARAT therapy (either enzalutamide or abiraterone), pre-specified by investigator, if randomized to Treatment Arm B. | | |
| | | | 12. ECOG performance status 0 to 1. | | |
| | | | 13. Willing and able to comply with all study requirements and treatments (including $^{177}$Lu-PNT2002) as well as the timing and nature of required assessments. | | |
| | | | 14. Signed informed consent. | | |

**<u>Exclusion Criteria</u>**
(**Reminder:** All answers must be answered No or N/A for the subject to be eligible.)

| Yes | No | N/A | Exclusion Criteria | Criteria Answer | Date Criteria Completed |
|---|---|---|---|---|---|
| | | | 1. If noted in pathology report, prostate cancer with known significant (>10% present in cells) sarcomatoid or spindle cell or neuroendocrine components. Any small cell component in the cancer should result in exclusion. | | |
| | | | 2. Prior treatment for prostate cancer ≤28 days prior to randomization, with the exclusion of first-line local external beam, ARAT, luteinizing hormone-releasing hormone (LHRH) agonist or antagonist therapy, or non-radioactive bone-targeted agents. | | |
| | | | 3. Any prior cytotoxic chemotherapy for CRPC (e.g., cabazitaxel or docetaxel); chemotherapy for hormone sensitive prostate cancer (HSPC) is allowed if the last dose was administered >1 year prior to consent. | | |
| | | | 4. Prior treatment with systemic radionuclides (e.g. radium-223, rhenium-186, strontium-89). | | |
| | | | 5. Prior immuno-therapy, except for sipuleucel-T. | | |
| | | | 6. Prior PSMA-targeted radioligand therapy, e.g., Lu-177-PSMA-617, I 131-1095. | | |
| | | | 7. Prior poly ADP ribose polymerase (PARP) inhibitor for prostate cancer. | | |
| | | | 8. Patients who progressed on 2 or more lines of ARATs. | | |
| | | | 9. Patients receiving bone-targeted therapy (e.g. denosumab, zoledronic acid) not on stable doses for at least 4 weeks prior to randomization. | | |

| | | | | | |
|---|---|---|---|---|---|
| | | | 10. Administration of an investigational agent ≤60 days or 5 half-lives, whichever is shorter, prior to randomization. | | |
| | | | 11. Major surgery ≤30 days prior to randomization. | | |
| | | | 12. Estimated life expectancy <6 months as assessed by the principal investigator. | | |
| | | | 13. Presence of liver metastases >1 cm on abdominal imaging. | | |
| | | | 14. A superscan on bone scan defined as a bone scan that demonstrates markedly increased skeletal radioisotope uptake relative to soft tissues in association with absent or faint genitourinary tract activity[71]. | | |
| | | | 15. Dose escalation or initiation of opioids for **cancer-related pain** ≤30 days prior to consent up to and including randomization. | | |
| | | | 16. Known presence of central nervous system metastases. | | |
| | | | 17. Contraindications to the use of planned ARAT therapy, [Ga-68] PSMA-11, [F-18] DCFPyL or [Lu-177] PNT2002 therapy, including but not limited to the following.<br><br>o Hypersensitivity to [Ga-68] PSMA-11, [F-18] -DCFPyL or [Lu-177] PNT2002 excipients (Diethylenetriaminepentaacetic acid (DTPA), Sodium ascorbate, Lascorbic acid, Sodium gentisate, HCl, Sodium hydroxide)<br><br>o Recent myocardial infarction or arterial thrombotic events (in the past 6 months) or unstable angina (in the past 3 months), bradycardia or left ventricular ejection fraction measurement of < 50%<br><br>o History of seizures in patients planned to receive enzalutamide | | |

| | | | | | |
|---|---|---|---|---|---|
| | | | 18. Active malignancy other than low grade non - muscle invasive bladder cancer and non melanoma skin cancer. | | |
| | | | 19. Concurrent illness that may jeopardize the patient's ability to undergo study procedures. | | |
| | | | 20. Serious psychological, familial, sociological, or geographical condition that might hamper compliance with the study protocol and follow up schedule. Patients that travel need to be capable of repeated visits even if they are on the control arm. | | |
| | | | 21. Symptomatic cord compression, or clinical or radiologic findings indicative of impending cord compression. | | |
| | | | 22. Concurrent serious (as determined by the investigator) medical conditions, including, but not limited to, New York Heart Association class III or IV congestive heart failure (see 12.1 Appendix 1), unstable ischemia, uncontrolled symptomatic arrhythmia, history of congenital prolonged QT syndrome, uncontrolled infection, known active hepatitis B or C, or other significant co morbid conditions that in the opinion of the investigator would impair study participation or cooperation. | | |

**Eligibility checklist completed by:**

Name: ______________________________________ Date________________

**All pre study assessments required at screening have been completed per the protocol and all eligibility requirements have been verified.**
**The subject was determined to be:**

**Eligible** **Not Eligible**

**Eligibility verified by:**

Investigator Signature: ______________________________ Date________________

Secondary Reviewer: ___________________________________ Date________________

| | |
|---|---|
| **Subject Sequence Number:** | |
| **Sequence Number Assigned By:** | |
| **Date Sequence Number Assigned:** | |

## 5.11. Prior and Concomitant Therapy

All treatments (medications, including over-the-counter or prescription medicines, vitamins, and/or herbal supplements, or medical procedures) that the patient completed within 30 days prior to informed consent are to be recorded in the eCRF, including the reason for use; dates of administration including start and end dates; and dosage information including dose and frequency.

## 5.12. Permitted/Prohibited Medications

### 5.12.1. Permitted Medications

The following medications are allowed on both arms:

- Patients without prior surgical castration must be taking and willing to continue taking LHRH analog treatment throughout the study.
- Pre-specified continued use of glucocorticoids to prevent secondary mineralocorticoid excess syndrome is permitted but should not be added after randomization unless prescribed as part of the ARAT regimen for Arm B.
- Pre-specified bisphosphonates or denosumab is permitted provided the dose is stable and started at least 4 weeks prior to study treatment but should not be started after randomization.
- Palliative external beam radiation.
- Palliative surgical procedures to treat skeletal-related events.
- Dose escalation or initiation of opioids for cancer-related pain.

Investigator discretion should be used for any deviations from the guidelines above to ensure the patient’s safety. Deviations should be record [illegible] evaluation of continuing eligibility.

### 5.12.2. Prohibited Medications

The following medications are prohibited during participation in the study:

- Other investigational agents.
- Other systemic radioisotopes.
- Poly ADP ribose polymerase inhibitors.
- Cytotoxic chemotherapy.
- Hemi-body radiotherapy.
- For patients randomized to receive abiraterone:
    - Strong inducers of CYP3A4 (e.g., phenytoin, carbamazepine, rifampicin, rifabutin, rifapentine, phenobarbital, St John's wort [Hypericum perforatum]) during treatment are to be avoided.
    - Caution is advised when administering with medicinal products activated by or metabolized by CYP2D6. Examples of medicinal products metabolized by CYP2D6 include metoprolol, propranolol, desipramine, venlafaxine, haloperidol, risperidone, propafenone, flecainide, codeine, oxycodone and tramadol (the latter three medicinal products requiring CYP2D6 to form their active analgesic metabolites).

  - Caution is to be exercised when abiraterone is combined with medicinal products that are predominantly eliminated by CYP2C8, and patients should be monitored for signs of toxicity related to a CYP2C8 substrate if used concomitantly.
  - Abiraterone may increase the concentrations of medicinal products eliminated by OATP1B1.
  - Since androgen deprivation treatment may prolong the QT interval, caution is advised when administering abiraterone with medicinal products known to prolong the QT interval or medicinal products able to induce torsades de pointes such as class IA (e.g. quinidine, disopyramide) or class III (e.g. amiodarone, sotalol, dofetilide, ibutilide) antiarrhythmic medicinal products, methadone, moxifloxacin, antipsychotics, etc.
  - Spironolactone binds to the androgen receptor and may increase prostate specific antigen (PSA) levels. Use with abiraterone is not recommended
  - Caution is to be exercised with drugs known to be associated with myopathy/rhabdomyolysis.
- For patients randomized to receive enzalutamide:
  - Enzalutamide is considered a strong inducer of CYP3A4 and a moderate inducer of CYP2C9 and CYP2C19.
  - Substrates of CYP3A4, CYP2C9, and CYP2C19 with a narrow therapeutic indices should be avoided. If enzalutamide is coadministered with warfarin or acenocourmarol (CYP2C9 substrate), additional international normalized ratio (INR) monitoring should be conducted.
  - Strong CYP2C8 inhibitors such as gemfibrozil should be avoided

[redacted] [redacted]

[redacted] [redacted]

[redacted]

[redacted] [redacted]

[redacted]

- [redacted]
  - [redacted]
  - [redacted]
- [redacted]

# STUDY SUBJECT ELIGIBILITY CHECKLIST

| | |
|---|---|
| **VICC Number and Study Title** | **MD22100**: A Study using StrataEXP to Identify RNA-Expression Biomarkers in Advanced Cancer Patients "StrataEXPress$^{TM}$" |
| **Protocol Version & Date** | **Version 0, 13 December 2021** |
| **Subject Initials** (First, Middle, Last) | |
| **Subject Medical Record Number** | |
| **Subject Date of Birth** | |

Patients must meet all of the following inclusion and exclusion criteria to be eligible for participation in this study. Refer to protocol to verify baseline test.

## Inclusion Criteria

(**Reminder:** All answers must be answered Yes or N/A for the subject to be eligible.)

| Yes | No | N/A | Inclusion Criteria | Criteria Answer | Date Criteria Completed |
|---|---|---|---|---|---|
| ☐ | ☐ | ☐ | 1. Must be ≥18 years of age, | | |
| ☐ | ☐ | ☐ | 2. Participant must have pathologically confirmed advanced, metastatic, or recurrent solid tumor, | | |
| ☐ | ☐ | ☐ | 3. Measurable disease, | | |
| ☐ | ☐ | ☐ | 4. Eastern Cooperative Oncology Group (ECOG) performance status score of 0-2, | | |
| ☐ | ☐ | ☐ | 5. Participant must meet at least 1 of the following:<br>a. Is/has not adequately responded to standard therapy, or<br>b. For whom no life-extending standard therapy exists, or<br>c. Who decline standard therapy, or<br>d. In the opinion of the investigator, is not a candidate for or would be unlikely to | | |

| | | | | | |
|---|---|---|---|---|---|
| | | | tolerate or derive significant clinical benefit from standard therapy | | |
| ☐ | ☐ | ☐ | 6. Adequate cardiac, bone marrow, organ function & laboratory parameters as determined by the treating physician for potential participation in a clinical trial, | | |
| ☐ | ☐ | ☐ | 7. Leftover formalin-fixed, paraffin-embedded (FFPE) tumor tissue available for StrataEXP Testing | | |

**Exclusion Criteria**

(**Reminder:** All answers must be answered No or N/A for the subject to be eligible.)

| **Yes** | **No** | **N/A** | **Exclusion Criteria** | **Criteria Answer** | **Date Criteria Completed** |
|---|---|---|---|---|---|
| ☐ | ☐ | ☐ | 1. Females who are pregnant or nursing, | | |
| ☐ | ☐ | ☐ | 2. History of stroke including transient ischemic attack (TIA) or acute myocardial infarction within 4 months of enrollment, | | |
| ☐ | ☐ | ☐ | 3. Any other clinically significant medical condition that, in the opinion of the treating physician, makes participation in a clinical trial undesirable, including but not limited to ongoing or active infection, significant uncontrolled hypertension, or severe psychiatric illness. | | |

**Eligibility checklist completed by:**

Name: ______________________________________ Date________________

**All pre-study assessments required at screening have been completed per the protocol and all eligibility requirements have been verified**.

**The subject was determined to be:**

**Eligible** **Not Eligible**

**Eligibility verified by:**

Investigator Signature: ______________________________ Date_______________

Secondary Reviewer: ___________________________________ Date_______________

---

| | |
|---|---|
| **Subject Sequence Number:** | |
| **Sequence Number Assigned By:** | |
| **Date Sequence Number Assigned:** | |

ACADEMIC AND COMMUNITY CANCER RESEARCH UNITED
(ACCRU)

Eligibility Checklist



**ACCRU-GI-2017: A Phase II study of Nivolumab + Ipilimumab in Advanced HCC Patients Who Have Progressed on First Line Atezolizumab + Bevacizumab**

***To register a patient, access the ACCRU web page at www.accru.org, go to the Application section and click*** on “Registration” ***and enter the registration/randomization application.***

Registration date (date on) *(mm/dd/yyyy)* __ __/__ __/__ __ __ __

Patient study ID number *(provided at Registration)* ______________________________

ACCRU member (participant sponsor) ______________________________

ACCRU treating location ______________________________

ACCRU treating physician/health care professional ______________________________

Institution patient number (local subject number) ______________________________

Person Completing Form:

Last Name: **(print)** ______________________ First Name: **(print)** ______________________

Phone: ______________ Fax: ______________ Email: ______________________

Patient initials *(last, first, middle)* ____ ____ ____

Gender *(check one)* ☐ Male ☐ Female ☐ Unknown ☐ Intersex

Date of birth *(mm/dd/yyyy)* __ __/__ __/__ __ __ __

ZIP code ______________________

Country of Residence ______________________

MedDRA disease code ______________________

Method of payment *(check one)*

- ☐ Private Insurance *(PI)*
- ☐ Medicare *(MR)*
- ☐ Medicare and Private Insurance *(MRP)*
- ☐ Medicaid *(MD)*
- ☐ Medicaid and Medicare *(MM)*
- ☐ Military or Veterans Sponsored, Not Otherwise Specified (NOS) *(MVA)*
- ☐ Military Sponsored [including CHAMPUS & TRICARE]*(MS)*
- ☐ Veterans Sponsored *(VS)*
- ☐ Self pay [no insurance] *(SP)*
- ☐ No means of payment [no insurance] *(NP)*
- ☐ Other *(OTH)*
- ☐ Unknown *(UNK)*

Race *(check all that apply)*

- ☐ White
- ☐ Black or African American
- ☐ Native Hawaiian or Other Pacific Islander
- ☐ Asian
- ☐ American Indian or Alaska Native
- ☐ Not reported *(Patient refused or not available)*
- ☐ Unknown *(Patient unsure)*

Ethnicity *(check one)*

- ☐ Not Hispanic or Latino
- ☐ Hispanic or Latino
- ☐ Not reported *(Refused or data not available)*
- ☐ Unknown *(Unsure of their ethnicity)*

Patient study ID number ________________________

Eligibility Check –Answer questions below (yes/no). All requirements must be confirmed. All dates are to be *mm/dd/yyyy*.

**Inclusion Criteria**

| Criterion | Yes | No | NA |
|---|---|---|---|
| Age ≥18 years. Age = _________. | ☐ | ☐ | |
| HCC diagnosis confirmed by histology/cytology or clinically by American Association for Study of Liver Diseases (AASLD)[31] criteria in cirrhotic patients. | ☐ | ☐ | |
| Measurable disease as defined by RECIST v1.1 (See Section 11.0). | ☐ | ☐ | |
| Locally advanced, metastatic, or unresectable disease. | ☐ | ☐ | |
| Child Pugh Class A. | ☐ | ☐ | |
| BCLC Stage B (not amenable to liver directed therapy) or Stage C. | ☐ | ☐ | |
| Prior treatment with atezolizumab and bevacizumab combination with radiographic progression that necessitates change in treatment per treating physician. Patients with rapid progression on atezolizumab and bevacizumab (defined as patients who progress in the first 6 weeks following initiati[illegible]) are excluded. | ☐ | ☐ | |
| Washout period ≥4 weeks prior to registration is required since last atezolizumab and bevacizumab dose. | ☐ | ☐ | |
| ECOG Performance Status (PS) 0 or 1 (Form is available on the ACCRU web site).<br>ECOG Performance Status = ________. | ☐ | ☐ | |
| The following laboratory values obtained  28 days prior to registration<br>Earliest laboratory test date __ __/__ __/__ __ __ __; latest laboratory test date __ __/__ __/__ __ __ __.<br>NOTE: These dates pertain to the following labs only.<br>NOTE: If your site laboratory reports use different units of measurements than what is required by the protocol eligibility requirements, please use the "Lab [illegible] under "General Forms". | ☐ | ☐ | |
| • Absolute neutrophil count (ANC) ≥1000/mm³. ANC = ________. | ☐ | ☐ | |
| • Platelet count ≥60,000/mm³. Platelet count = ________. | ☐ | ☐ | |
| • Hemoglobin ≥8.5 g/dL. Hemoglobin = ________. | ☐ | ☐ | |
| • Total bilirubin ≤ 3 x upper limit of normal (ULN). Total bilirubin = ________; ULN = ________. | ☐ | ☐ | |
| • Alanine aminotransferase (ALT) and aspartate transaminase (AST)  5 x ULN<br>ALT (≤ 5 x ULN) = _______; ULN = _______.<br>AST (≤ 5 x ULN) = _______; ULN = _______. | ☐ | ☐ | |
| • INR ≤2.3 or Prothrombin time (PT) ≤ 6 second[illegible] therapy and INR is within target range of therapy<br>**Is patient receiving anticoagulant therapy and INR is within target range of therapy?**<br>☐ Yes, Proceed to next question.<br>☐ No **Which test was done to satisfy eligibility?**<br>☐ INR (≤ 2.3) = ______<br>☐ PT (≤ 6) = ______ | ☐ | ☐ | |
| • Creatinine ≤1.5x ULN<br>Creatinine = ________; ULN = _______. | ☐ | ☐ | |
| Negative serum pregnancy test done  7 days prior to registration, for women of childbearing potential only.<br>NOTE: If the urine test is positive or cannot be confirmed as negative, a serum pregnancy test will be required.<br>If not a woman of childbearing potential *(check NA)*<br>If a woman of childbearing potential –Negative serum pregnancy test date __ __/__ __/__ __ __ __ | ☐ | ☐ | ☐ |
| Provide informed written consent ≤28 days prio[illegible]<br>Date informed consent signed __ __/__ __/__ __ __ __ | ☐ | ☐ | |

Patient study ID number ________________________

**Inclusion Criteria** –*(Continued)*

| | Yes No NA |
|---|---|
| Willing to return to enrolling institution for follow-up (during the Active Monitoring Phase of the study).<br>Note: During the Active Monitoring Phase of a study (i.e., active treatment), participants must be willing to return to the consenting institution for follow-up. | ☐ ☐ |
| Willing to provide mandatory tissue specimens and blood specimens for correlative research purposes (see Sections 6.0, 14.0 and 17.0). | ☐ ☐ |

All responses in above section must be “Yes” unless specif

**Exclusion Criteria**

| | Yes No NA |
|---|---|
| Any of the following because this study involves an investigational agent whose genotoxic, mutagenic and teratogenic effects on the developing fetus and newborn are unknown<br>• Pregnant persons<br>• Nursing persons<br>• Persons of childbearing potential who are unwilling to employ adequate contraception | ☐ ☐ |
| Major surgery ≤4 weeks prior to registration.<br>If no prior major surgery *(check NA)*<br>If prior major surgery –Date of surgery __ __/__ __/__ __ __ __ | ☐ ☐ ☐ |
| Liver directed therapy (TACE, Y-90, liver directed radiation) ≤ 28 days prior to reg therapy >28 days prior to registration is allowed as long as patient has at least one measurable untreated lesion by RECIST v1.1<br>If no prior liver directed therapy *(check NA)*<br>If prior liver directed therapy –Date of therapy __ __/__ __/__ __ __ __ | ☐ ☐ ☐ |
| Patients with rapid progression on atezolizumab and bevacizumab (patients who progress in the first 6 weeks following initiation of therapy) are excluded. | ☐ ☐ |
| Prior treatment ≤ 4 weeks prior to registration with anti-CTLA-4 antibody for HCC.<br>If no prior treatment with anti-CTLA-4 antibody for HCC *(check NA)*<br>If prior treatment with anti-CTLA-4 antibody for HCC –Last day of therapy __ __/__ __/__ __ __ __ | ☐ ☐ ☐ |
| Co-morbid systemic illnesses or other severe concurrent disease which, in the judgment of the investigator, would make the patient inappropriate for entry into this study or interfere significantly with the proper assessment of safety and toxicity of the prescribed regimens. | ☐ ☐ |
| Immunocompromised patients and patients known to be HIV positive and currently receiving antiretroviral therapy.<br>NOTE: Patients known to be HIV positive, but without clinical evidence of an immunocompromised state, are eligible for this trial. | ☐ ☐ |
| Uncontrolled intercurrent illness including, but not limited to:<br>• ongoing or active infection excluding HCV<br>• symptomatic congestive heart failure<br>• unstable angina pectoris<br>• unstable cardiac arrhythmia<br>• psychiatric illness/social situations that would limit compliance with study requirements.<br>• Active infection including tuberculosis (clinical evaluation that includes clinical history, physical examination and radiographic findings, and TB testing in line with local practice), hepatitis B (known positive HBV surface antigen (HBsAg) result). Patients with a past or resolved HBV infection (defined as the presence of hepatitis B core antibody [anti-HBc] and absence of HBsAg) are eligible. Patients with chronic HBV infection as evidenced by detectable HBV surface antigen or HBV DNA are eligible if on antiviral therapy and have HBV DNA <100 IU/mL. Patients with active or resolved hepatitis C (HCV) infection as evidenced by detectable HCV RNA or antibody are eligible. | ☐ ☐ |
| Receiving any other investigational agent which would be considered as a treatment for the primary neoplasm ≤4 weeks prior to registration.<br>If no prior treatment with investigational agent considered treatment for primary neoplasm *(check NA)*<br>If prior treatment with investigational agent –Last day of treatment __ __/__ __/__ __ __ __ | ☐ ☐ ☐ |

Patient study ID number ________________________

**Exclusion Criteria –***(continued)*

| | Yes | No | NA |
|---|---|---|---|
| Other active malignancy ≤2 years prior to registration.<br>EXCEPTIONS: Non-melanotic skin cancer or carcinoma-in-situ of the cervix. | ☐ | ☐ | |
| History of myocardial infarction ≤6 months, or congestive heart failure requiring use of ongoing maintenance therapy for life-threatening ventricular arrhythmias. | ☐ | ☐ | |
| History of allogenic organ transplantation. | ☐ | ☐ | |
| Active or prior documented autoimmune or inflammatory disorders (including inflammatory bowel disease [e.g., colitis or Crohn's disease], diverticulitis [with the exception of diverticulosis], systemic lupus erythematosus, Sarcoidosis syndrome, or Wegener syndrome [granulomatosis with polyangiitis, Graves' disease, rheumatoid arthritis, hypophysitis, uveitis, etc]). The following are exceptions to this criterion:<br>• Patients with vitiligo or alopecia<br>• Patients with hypothyroidism (e.g., following Hashimoto syndrome) stable on hormone replacement<br>• Any chronic skin condition that does not require systemic therapy<br>• Patients without active disease in the last 5 years may be included but only after consultation with the study physician<br>• Patients with celiac disease controlled by diet alone | ☐ | ☐ | |
| History of leptomeningeal carcinomatosis | ☐ | ☐ | |
| Known allergy or hypersensitivity to any of the study drugs or any of the study drug excipients. | ☐ | ☐ | |
| Current or prior use of immunosuppressive medication ≤14 days prior to registration. The following are exceptions to this criterion:<br>• Intranasal, inhaled, topical steroids, or local steroid injections (e.g., intra articular injection)<br>• Systemic corticosteroids at physiologic doses not to exceed 10 mg/day of prednisone or its equivalent<br>• Steroids as premedication for hypersensitivity reactions (e.g., CT scan premedication)<br>If no prior use of immunosuppressive medication (check NA)<br>If prior use of immunosuppressive medication (excluding exceptions listed above) –<br>Last day of immunosuppressive medication use ___/___/______ | ☐ | ☐ | ☐ |
| Receipt of live attenuated vaccine ≤30 days pr [illegible], if enrolled, should not receive live vaccine whilst on study treatment and up to 30 days after the last dose of study treatment.<br>If no prior receipt of live attenuated vaccine (check NA)<br>If prior receipt of live attenuated vaccine –Date of vaccine ___/___/______ | ☐ | ☐ | ☐ |
| Any unresolved toxicity NCI CTCAE v5.0 Grade ≥2 from previous antica [illegible] alopecia, vitiligo, and the laboratory values defined in the inclusion criteria. | ☐ | ☐ | |
| History of Grade 3 or 4 immunotherapy related toxicity NCI CTCAE V5 due to prior regimen attributed to atezolizumab. | ☐ | ☐ | |

All responses in "**No**" column unless otherwise specified as "NA."

Patient study ID number ______________________

Registration Check –Answer questions below (yes/no). All requirements must be confirmed. All dates are to be *mm/dd/yyyy*.

| | Yes | No | NA |
|---|---|---|---|
| A mandatory correlative research component is part of this study. The patient will be automatically registered onto this component (see Sections 3.0, 14.0 and 17.0). | ☐ | ☐ | |
| Consent form signed and dated. | ☐ | ☐ | |
| Existence of authorization for use and disclosure of protected health information. | ☐ | ☐ | |
| Treatment cannot begin prior to registration and must begin 14 days after randomization. | ☐ | ☐ | |
| Pretreatment tests/procedures (see Section 4.0) must be completed 28 days prior to randomization.<br>Earliest pretreatment test date __ __/__ __/__ __ __ __; latest pretreatment test date __ __/__ __/__ __ __ __ | ☐ | ☐ | |
| All required baseline symptoms (see Section 10.5) must be documented and graded. | ☐ | ☐ | |
| Treatment on this protocol must commence at an ACCRU institution under the supervision of a medical oncologist. | ☐ | ☐ | |
| Study drug is available on site. | ☐ | ☐ | |
| Blood draw kit is available on site. | ☐ | ☐ | |

All responses in above section must be "Yes"

Registration Check –*continued*

| | Yes | No |
|---|---|---|
| At the time of registration, the following will be recorded:<br>• Patient has given permission to store and use his/her sample(s) for future research to learn about, prevent, or treat cancer. | ☐ | ☐ |
| • Patient has given permission to store and use his/her sample(s) for future research to learn, prevent, or treat other health problems (for example: d | ☐ | ☐ |
| • Patient has given permission for ACCRU to give his/her sample(s) to outside researchers. | ☐ | ☐ |

All responses in above section may be "Yes" or "No".

Assigned Treatment

Nivolumab + Ipilimumab

Person registering Signature ____________________________ Registration Office specialist initials _______

Physician Signature ___________________________________ Date *(mm/dd/yyyy)* __ __/__ __/__ __ __ __

| Subject Eligibility Accompanying Form & Review Form<br>Protocol V4.0 dated 14Sep2023 | |
|---|---|
| Site #: | Subject #: |
| Site Name: | Subject Year of Birth: |
| Anticipated Treatment Start Date (C1D1): | Date of Informed Consent: |
| PI Name: | Date: |

<u>This form is being completed by sites where source documents cannot be sent outside the hospital, due to institutional or local regulations. This form is therefore being submitted in place of de-identified supporting documentation, in order for the Arcus Medical Monitor/Clinical Scientist to review eligibility.</u>

- Please complete and sign this Subject Eligibility Accompanying Form, enclose it with the Subject Enrollment Form and submit to *HAB74808-Faxes@iqvia.com* at least 48 hours prior to dosing. Copy your assigned CRA. Only page 1 of the Subject Enrollment Form is required to be completed if the Subject Eligibility Accompanying Form is utilized.
- Please ensure to send the requested documents via encrypted emails only. If using Outlook see instructions below:

***How to send an encrypted email from Outlook (PC)***

- *Click New Email.*
- *Click the Options toolbar.*
- *On the Options toolbar, click the Encrypt menu.*
- *On the Encrypt menu, click Encrypt-Only.*
- *Your new email is now encrypted.*

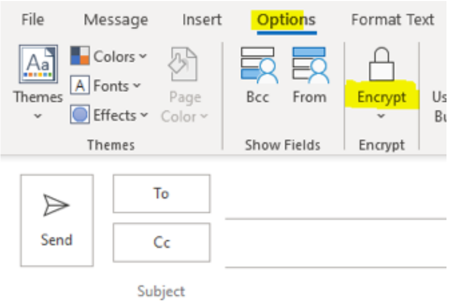


- *If you are unable to send documents via encrypted email, please contact the IQVIA Biotech Study Team via HAB74808-Faxes@iqvia.com.*
- Please submit the form at least 48 hours prior to the first planned dose.

| Site #: | PI: | Subject ID: |
|---|---|---|
| | | |

1. Past Medical history: None; Yes, please complete below

| Medical History Term | *Start Date (DD/MMM/YYYY)* | *End Date (DD/MMM/YYYY) or Ongoing* |
|---|---|---|
| | | |
| | | |
| | | |
| | | |
| | | |
| | | |
| | | |
| | | |

2. Current concomitant medications: None; Yes, please complete below

| Medication Name | Medication Details (e.g. Dose, Frequency, route of administration etc.) | Start Date *(DD/MMM/YYYY)* | End Date *(DD/MMM/YYYY) or Ongoing* |
|---|---|---|---|
| | | | |
| | | | |
| | | | |
| | | | |
| | | | |
| | | | |
| | | | |
| | | | |

| | **INCLUSION CRITERIA** | |
|---|---|---|
| 1. | Capable of giving signed informed consent, which includes compliance with the requirements and restrictions listed in the ICF and in this protocol. | ☐ Yes ☐ No |
| 2. | Male or female participants ≥ 18 yea regionally approved age of consent for participation in investigational clinical studies) at the time of signing the ICF. | ☐ Yes ☐ No |
| | If yes, Patient Year of Birth: | |
| 3. | Disease-specific criteria for <u>**dose escalation**</u><br>a. Participants may have any pathologically confirmed solid tumor type where no other treatment options are available, and<br>b. Creatinine clearance ≥ 40 mL/min as determined by -Gault equation (Cockcroft and Gault 1976) or other acceptable method. | ☐ Yes ☐ No ☐ NA |
| | **a.) Pathology Report Findings:**<br><br>Date:<br><br>solid tumor type/Interpretation: | |

| Site #: | PI: | Subject ID: |
|---|---|---|

**INCLUSION CRITERIA**

| | | |
|---|---|---|
| | b.) Creatinine Clearance: __________ mL/min<br><br>**Please use the equation: Cockcroft-Gault CrCl, mL/min =*<br>*(140 – age) × (weight, kg) × (0.85 if female) / (72 × Cr, mg/dL)* | |
| 4. | Disease-specific criteria for **dose-expansion**<br>a. Histologically confirmed ccRCC and,<br>b. Must have received prior treatment in the metastatic setting (either individually or in combination) with an anti-PD-1/PD-L1 therapy and a TKI, and<br>c. No prior treatment with an HIF-2α-targeting therapy.<br>d. Creatinine clearance ≥40 mL/min as determined by Cockcroft-Gault equation (Cockcroft and Gault 1976) or other acceptable method. | ☐ Yes ☐ No ☐ NA |
| | a.) Histologically confirmed clear cell RCC: Yes No<br>Date (s) of report (s) (dd-mmm-yyyy): ________________________<br><br>Pathology report summary:<br><br>b.) Prior anti-PD1/PD-L1 and TKI treatment in metastatic setting: | |

| Treatment given | Start Date (dd/mmm/yyyy) | End Date (dd/mmm/yyyy) |
|---|---|---|
| | | |
| | | |
| | | |

| | | |
|---|---|---|
| | c.) Prior HIF-2α-targeting therapy: Yes No<br>d.) Creatinine Clearance: __________ mL/min<br>**Please use the equation: Cockcroft-Gault CrCl, mL/min =*<br>*(140 – age) × (weight, kg) × (0.85 if female) / (72 × Cr, mg/dL)* | |
| 5. | Must have at least 1 measurable lesion per RECIST guidance (Version 1.1; see Appendix 5 in Section 10.5). | ☐ Yes ☐ No |
| | **Radiology Report Findings:**<br><br>Radiologic Modality used: CT MRI Other: ______________<br><br>Date:<br><br>Measurable lesions per RECIST:<br>Location: ________<br>Tumor lesion size, please indicate the longest axis (cm):<br>_________________<br><br>Location: ________<br>Tumor lesion size, please indicate the longest axis (cm):<br>_________________<br><br>Location: ________<br>Tumor lesion size, please indicate the longest axis (cm):<br>_________________ | |

| Site #: | PI: | Subject ID: |
|---|---|---|
| | | |

| INCLUSION CRITERIA | | |
|---|---|---|
| | Location: _______<br>Tumor lesion size, please indicate the longest axis (cm):<br>________________<br><br>Location: _______<br>Tumor lesion size, please indicate the longest axis (cm):<br>________________ | |
| 6. | Eastern Cooperative Oncology Group (ECOG) performance status score of ≤1 (see Appendix 4 in Section 10.4). | ☐ Yes ☐ No |
| | If Yes, ECOG performance Score 0 1 | |
| 7. | Negative tests for hepatitis B surface antigen and hepatitis C virus antibody (or hepatitis C qualitative RNA [qualitative]) at screening. COVID-19 negative test as required by regional policy or site standard operating procedures (SOPs). Note: Participants with treated hepatitis B and/or C with no evidence of active infection may be enrolled. | ☐ Yes ☐ No |
| | HBsAg Date (dd/mmm/yyyy): ___/___/___ Result: ______________________<br>(include normal range and units)<br>• If HbsAg testing is positive, please confirm: HBV<br>(dd/mmm/yyyy) _ _/____/___ Result: ☐ Negative ☐ Positive<br><br>HCV Ab Serology Date (dd/mmm/yyyy): ___/___/___ Result: _____________<br>(include normal range and units)<br>• If HCV Ab testing is positive, please confirm: HCV RNA Date<br>(dd/mmm/yyyy) ___/____/___ Result: ☐ Negative ☐ Positive | |
| 8. | Adequate organ and marrow function including:<br>a. Absolute neutrophil count (ANC) ≥1.5 × $10^{9}$/L<br>b. Platelet count ≥100 × $10^{9}$/L<br>c. Hemoglobin ≥10.0 g/dL (or 6.2 mmol/L)<br>d. Aspartate transaminase (AST) ≤ 2.5 × ULN without hepatic metastasis and ≤ 5 × ULN with hepatic metas<br>e. Alanine aminotransferase (ALT) ≤ 2.5 × ULN withou metastasis and ≤ 5 × ULN with hepatic metastasis<br>f. Bilirubin ≤ 1.5 × ULN (except participants with G syndrome who must have total bi | ☐ Yes ☐ No |
| | **Lab Values:**<br><br>a.) Absolute neutrophil count (ANC): _________ unit:___________<br>b.) Platelet count : _________ unit:___________<br>c.) Hemoglobin: _________ unit:___________<br>d.) Aspartate transaminase (AST): _________ unit:___________<br>e.) Alanine aminotransferase (ALT): _________ unit:___<br>f.) Bilirubin: _________ unit:___________ | |
| 9. | Screening ambulatory oxygen saturation ($SpO_2$) ≥ 92%. | ☐ Yes ☐ No |
| | | |

| Site #: | PI: | Subject ID: |
|---|---|---|
| | | |

| INCLUSION CRITERIA | | |
|---|---|---|
| | If Yes, $SpO_2$ value: _________ | |
| 10 | No radiological evidence of pneumonitis at screening. | ☐ Yes ☐ No |
| 11 | No medical history of severe chronic obstructive pulmonary disease (COPD). | ☐ Yes ☐ No |
| 12 | No evidence of clinically significant pericardial effusion in screening echocardiogram or multigated acquisition scan (MUGA) | ☐ Yes ☐ No |
| 13 | Contraceptive use by males and females should be consistent with local regulations regarding the methods of contraception for those participating in clinical studies.<br>a. Male participants must use effective contraceptive methods during treatment and for 90 days post last dose and refrain from donating sperm during treatment and for 90-days post last dose (see Section 10.2 for additional information).<br>b. Given the potential effect on some hormonal contraceptives, females of childbearing potential who are using hormonal contraceptives must be willing to use a second effective non-hormonal method of contraception (eg, male condom, diaphragm with spermicide). | ☐ Yes ☐ No |
| 14 | A female participant is eligible to participate if she is not pregnant (Section 10.2), not breastfeeding, not planning to become pregnant, and at least one of the following conditions applies:<br>a. Not a woman of childbearing potential (WOCBP) as defined in Appendix 2 (Section 10.2)<br>OR<br>b. A WOCBP who agrees to follow the contraceptive guidance in Appendix 2 (Section 10.2) during the treatment period and for 90 days post last dose and agrees to not donate ova during the treatment period and for 90 days post last dose. | ☐ Yes ☐ No<br>☐ NA - Male |
| 15 | Willing and able to comply with the requirements and restrictions in this protocol. | ☐ Yes ☐ No |

| EXCLUSION CRITERIA | | |
|---|---|---|
| 1. | Use of live vaccines against infectious diseases (eg, influenza, varicella) within 4 weeks (28 days) of initiation of investigational product. | ☐ Yes ☐ No |
| 2. | Underlying medical cond[illegible]'s judgment, will make[illegible], the administration of investigational product hazardous (eg, interstitial lung disease, active infections requiring antibiotics, recent hospitalizations with unresolved symptoms) or obscure the interpretation of toxicity determination or AEs. | ☐ Yes ☐ No |
| 3. | History of trauma or major surgery within 28 days prior to the first dose of investigational product. (Note that placement of central venous access catheter [eg, port or similar] is not considered a major surgical procedure). | ☐ Yes ☐ No |
| 4. | For monotherapy dose expansion: prior treatment with an HIF-2α inhibitor. | ☐ Yes ☐ No ☐ NA |
| 5. | Has known psychiatric or substance abuse disorders that would interfere with cooperation with the requirements of the trial. | ☐ Yes ☐ No |
| 6. | Positive serum pregnancy test at screening and prior to dosing on Cycle 1 Day 1 (WOCBP only). | ☐ Yes ☐ No<br>☐ NA – Male or Not WOCBP |
| | Serum Pregnancy Result (if applicable): Positive Negative | |

| Site #: | PI: | Subject ID: |
|---|---|---|

| EXCLUSION CRITERIA | | |
|---|---|---|
| | | |
| 7. | Is pregnant or breastfeeding or trying to conceive or father children within the projected duration of the study, starting with the pre-screening or screening visit through 90 days after the last dose of study treatment. | ☐ Yes ☐ No |
| 8. | Other prior malignancy active within the previous year except for locally curable cancers that have been apparently cured, such as basal or squamous cell skin cancer, superficial bladder cancer, or carcinoma in situ of the cervix, breast, or prostate cancer. Also, indolent malignancies, including but not limited to early-stage chronic lymphocytic leukemia and follicular lymphoma, that don’t require anti-cancer treatment, could be allowed after discussion with the medical monitor. | ☐ Yes ☐ No |
| 9. | Prior chemotherapy, targeted small-molecule therapy, immunotherapy (tumor vaccine, cytokine, or growth factor given to control the cancer), or biologic agents, or use of other investigational drugs (drugs not marketed for any indication) within 28 days or 5 half-lives (whichever is shorter) before investigational product administration. | ☐ Yes ☐ No |
| | Last Treatment: / End Date (dd/mmm/yyyy) | |
| 10. | QTc ≥480 msec using Fredericia’s correction (QTcF) (based on an average of triplicate recordings) | ☐ Yes ☐ No |
| | QTc result: ____________ msec | |
| 11. | Known hypersensitivity to any excipient contained in the IP formulations. | ☐ Yes ☐ No |
| 12. | Inability to swallow study treatment. | ☐ Yes ☐ No |
| 13. | Malabsorption condition that would alter the absorption of orally administered medications | ☐ Yes ☐ No |

| Last Treatment: | End Date (dd/mmm/yyyy) |
|---|---|
| | |

Site Comments / Additional Information:

| Site #: | PI: | Subject ID: |
|---|---|---|
| | | |

| FORM COMPLETED | |
|---|---|
| Name of person completing this form: ______________________________ | Date: ____________ |

## Principal Investigator or Sub Investigator Confirmation*

| Name (Print) | |
|---|---|
| Signature | Date |

*Confirmation of eligibility must be signed by the Principal Investigator or Sub-Investigator

## Arcus Medical Monitor/Clinical Scientist Review

| Date Subject eligibility criteria has been reviewed: | |
|---|---|
| Medical Monitor/Clinical Scientist comments: | |
| Name (Print) | |
| Signature | Date |

## SUBJECT ELIGIBILITY CHECKLIST

| | |
|---|---|
| **CLIENT: SPHBio** | **PROTOCOL NUMBER: SPH4336-US-01** |
| **PRINCIPAL INVESTIGATOR (PI):** | **SITE NUMBER:** |
| | **SUBJECT NUMBER:** |

## SUBJECT ELIGIBILITY CHECKLIST SUBMISSION INSTRUCTIONS

- Upon the completion of all screening activi[illegible] eligibility, please complete this Subject Eligibility Checklist and email to Precision at *SPH4336-US-01_Study_Eligibility_Mailbox@precisionformedicine.com* within 48 hours of planned dosing.
- Approval to enroll will be sent to you by the Clinical Trial Manger, or designee, via email within 24 hours of planned dosing if received without any error.
- The fully completed and signed Subject Eligibility Checklist and email approval to enroll should be filed with the subject's source d[illegible]

| Inclusion Criteria | Criteria Verification |
|---|---|
| Informed consent | ☐ Yes<br>☐ No<br>Comment: |
| ≥18 years of age | ☐ Yes<br>☐ No<br>Comment: |
| ECOG performance status 0 or 1[1] | ☐ Yes<br>☐ No<br>Comment: |
| Histologically confirmed, locally advanced or metastatic sarcoma | ☐ Yes<br>☐ No<br>Comment: |
| ○ Dedifferentiated or well-differentiated/dedifferentiated liposarcomas | ☐ Yes<br>☐ No<br>Comment: |

PRECISION for medicine

SPHBio SHANGHAI PHARMA BIOTHERAPEUTICS USA INC

# SUBJECT ELIGIBILITY CHECKLIST

| | |
|---|---|
| **CLIENT: SPHBio** | **PROTOCOL NUMBER: SPH4336-US-01** |
| **PRINCIPAL INVESTIGATOR (PI):** | **SITE NUMBER:** |
| | **SUBJECT NUMBER:** |

| Inclusion Criteria | Criteria Verification |
|---|---|
| No more than 3 prior lines of treatment | ☐ Yes<br>☐ No<br>Comment: |
| Evidence of progression as evidenced by at least one of the following within the past 3 months:<br>o An increase of at least 20% in measurable tumors<br>o The appearance of new lesions<br>o Unequivocal progression of non-measurable lesions | ☐ Yes<br>☐ No<br>Comment: |
| Measurable disease per RECIST v1.1[2] | ☐ Yes<br>☐ No<br>Comment: |
| If residual treatment-related toxicity from prior therapy:<br>o All treatment-related toxicity resolved to Grade 1 or baseline (alopecia excepted) | ☐ Yes<br>☐ No<br>Comment: |
| ANC ≥ 1,500/μL | ☐ Yes<br>☐ No<br>Comment: |
| Platelets ≥ 100,000/μL | ☐ Yes<br>☐ No<br>Comment: |
| Hgb ≥ 9.0 g/dL (in the absen<br>4 weeks) | ☐ Yes<br>☐ No<br>Comment: |
| Estimated glomerular filtrat<br>Cockcroft and Gault formula for individualized estimates of GFR[3]) | ☐ Yes<br>☐ No<br>Comment: |



Subject Initials: ___________________

# SUBJECT ELIGIBILITY CHECKLIST

| | |
|---|---|
| **CLIENT: SPHBio** | **PROTOCOL NUMBER: SPH4336-US-01** |
| **PRINCIPAL INVESTIGATOR (PI):** | **SITE NUMBER:** |
| | **SUBJECT NUMBER:** |

| Inclusion Criteria | Criteria Verification |
|---|---|
| Total bilirubin ≤ 1.5 x the ULN if known Gilbert's disea | ☐ Yes<br>☐ No<br>Comment: |
| AST and ALT ≤ 3 x ULN or ≤ 5<br>of the liver | ☐ Yes<br>☐ No<br>Comment: |
| Sterile or willing to use effective contraception (approved hormonal contraceptive such as oral contraceptives, patches, implants, injections, rings or hormonally-impregnated intrauterine device (IUD), or an IUD in women of childbearing potential and a condom in men) during the study and for 3 months following the last dose of study drug | ☐ Yes<br>☐ No<br>Comment: |
| Availability of archived tumor tissue or willingness to undergo a baseline tumor biopsy, and in the first 10 study subjects, to determine baseline tumor biomarker levels and a willingness to undergo a second tumor biopsy at C1D15 to assess treatment-induced changes in tumor biomarker levels | ☐ Yes<br>☐ No<br>Comment: |

## SUBJECT ELIGIBILITY CHECKLIST

| CLIENT: SPHBio | PROTOCOL NUMBER: SPH4336-US-01 |
|---|---|
| PRINCIPAL INVESTIGATOR (PI): | SITE NUMBER: |
| | SUBJECT NUMBER: |

| Exclusion Criteria | Criteria Verification |
|---|---|
| Prior treatment with a CDK4/6-targeted agent | ☐ No<br>☐ Yes<br>Comment: |
| Patient's tumor known | ☐ No<br>☐ Yes<br>Comment: |
| Anticancer therapy (e.g., chemotherapy, biologics, irradiation) within 14 days or 5 half-lives (whichever is greater) of screening | ☐ No<br>☐ Yes<br>Comment: |
| Major surgery within 28 days of screening | ☐ No<br>☐ Yes<br>Comment: |
| Requirement for systemic treatment with strong CYP3A4 inhibitors or inducers of CYP3A4 at study entry | ☐ No<br>☐ Yes<br>Comment: |
| Central nervous system metastases or leptomeningeal disease, unless appropriately treated and neurologically stable without steroids for ≥ 28 days | ☐ No<br>☐ Yes<br>Comment: |
| Other malignancy unless disease-free for and not expected to relapse or require treatment during study participation | ☐ No<br>☐ Yes<br>Comment: |
| Active systemic infection or severe localized infection | ☐ No<br>☐ Yes<br>Comment: |

PRECISION for medicine

SPHBio SHANGHAI PHARMA BIOTHERAPEUTICS USA INC

# SUBJECT ELIGIBILITY CHECKLIST

| | |
|---|---|
| **CLIENT: SPHBio** | **PROTOCOL NUMBER: SPH4336-US-01** |
| **PRINCIPAL INVESTIGATOR (PI):** | **SITE NUMBER:** |
| | **SUBJECT NUMBER:** |

| Exclusion Criteria | Criteria Verification |
|---|---|
| Known HIV-positive with CD4+ cell counts < 350 cells/uL or a history of an AIDS-defining opportunistic infection | ☐ No ☐ Yes Comment: |
| Known hepatitis B virus (HBV) or hepatitis C virus (HCV) infection with viral load above the limit of quantification | ☐ No ☐ Yes Comment: |
| Active COVID-19 infection | ☐ No ☐ Yes Comment: |
| Major cardiac abnormalities (e.g., uncontrolled angina, unstable arrhythmias, myocardial infarction, NYHA Clas C1D1 | ☐ No ☐ Yes Comment: |
| Persistent (3 ECGs ≥ the QTcF (Fridericia) > 470 msec | ☐ No ☐ Yes Comment: |
| [Females] Pregnant or nursing | ☐ No ☐ Yes Comment: |
| Any other medical or psychiatric condition, or laboratory abnormality that would result in an unacceptable risk with study participation | ☐ No ☐ Yes mment: |
| Presence of active gastrointestinal disease or other condition expected to interfere significantly with absorption, distribution, metabolism or excretion of oral therapy (e.g., ulcerative disease, uncontrolled nausea, vomiting, chronic diarrhea, malabsorption syndrome) | ☐ No ☐ Yes Comment: |

## SUBJECT ELIGIBILITY CHECKLIST

| | |
|---|---|
| **CLIENT: SPHBio** | **PROTOCOL NUMBER: SPH4336-US-01** |
| **PRINCIPAL INVESTIGATOR (PI):** | **SITE NUMBER:** |
| | **SUBJECT NUMBER:** |

I have reviewed this subject's medical history and eligibility criteria and have determined that the subject meets the eligibility criteria for enrollment.

**Principal Investigator Signature: ________________________________** **Date: _____________**

## VANDERBILT SUBJECT ELIGIBILITY CHECKLIST SIGNATURE PAGE

| | |
|---|---|
| **VICC Number and Study Title** | **DTSAR23090:** A Phase 2 Multicenter, Open-Label Study of the CDK4/6 Inhibitor SPH4336 in Subjects With Locally Advanced or Metastatic Liposarcomas |
| **Protocol Version & Date** | **Protocol Version 3.0, dated September 01, 2023** |

### Eligibility checklist completed by:

Name: _______________________________________ Date________________

**All pre-study assessments required at screening have been completed per the protocol and all eligibility requirements have been verified**.

**The subject was determined to be:**

☐ **Eligible** ☐ **Not Eligible**

**Eligibility verified by:**

Investigator Signature: _______________________________ Date________________

Secondary Reviewer: ____________________________________ Date________________

---

| | |
|---|---|
| **Subject Sequence Number:** | |
| **Sequence Number Assigned By:** | |
| **Date Sequence Number Assigned:** | |
